# Acquired Trichorrhexis Nodosa Caused by Massage of the Scalp After Topical Minoxidil: A Case Report and Review of the Literature

**DOI:** 10.1111/jocd.70258

**Published:** 2025-06-30

**Authors:** Xi Chen, Xinzhou Liu, Jianxin Liu, Zhenbo Hu, Guoyan Liu

**Affiliations:** ^1^ Hospital for Skin Diseases Shandong First Medical University Jinan Shandong China; ^2^ Shandong Provincial Institute of Dermatology and Venereology Shandong Academy of Medical Sciences Jinan Shandong China; ^3^ Laboratory for Stem Cell and Regenerative Medicine Affiliated Hospital of Shandong Second Medical University Weifang Shandong China

**Keywords:** alopecia, baldness, stem cells

To the Editor,

1

Trichorrhexis nodosa (TN) is an abnormal hair shaft disease caused by hair breakage after nodular swelling. The clinical features included sparse, dry, and lusterless hair with dust‐like white spots. We present a patient diagnosed with acquired TN caused by excessive scalp massage after topical minoxidil. We advised the patient to correct the bad habits and gave a scalp injection of mesenchymal stem cell (MSC) exosomes as adjunctive therapy, which resulted in a rapid improvement of symptoms after treatment. A 47‐year‐old male presented with a decade‐long clinical history of alopecia (Figure 1A). He was started on topical 1 mL 5% minoxidil twice daily after he was diagnosed with androgenetic alopecia 3 months ago. Each application was followed by a careful 10 to 15‐min scalp massage to enhance absorption. Two months later, some hairs on the top of the head showed breakage. Three months later, round alopecia patches appeared on the front of the head, but the hair surrounding these patches became thicker than before treatment (Figure 1B). No other hair care practices are linked to an increase in mechanical damage.

**FIGURE 1:**
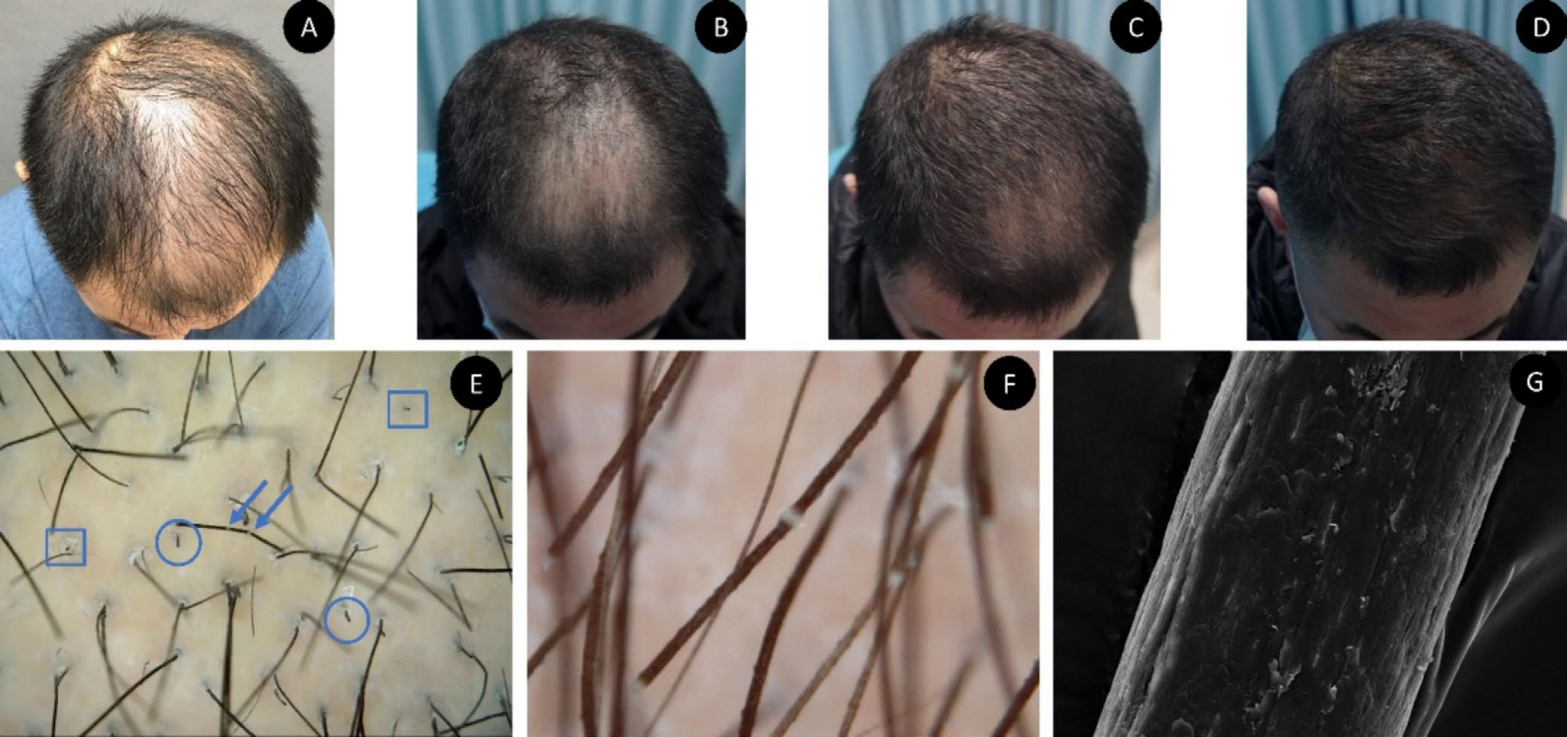
(A) Clinical photograph of the patient before treatment. (B) Round‐like alopecia visible on the top of the head. (C) Round‐like alopecia decreasing in size and hair lengthening. (D) Hair restoration in the alopecia area. (E) Dermoscopic view of the hair shaft showing multiple white nodules (blue arrows), broken hairs (blue circles), and the black dots sign (blue squares) (50×). (F) White nodules in the middle of the hair shaft with brush‐like changes at the ends (200×). (G) Scanning electron microscopy of part of the hair cuticle disappeared, and the residual hair cuticle was seen to be rough and rugged in the hair shaft.

Blood tests: There were no abnormalities in blood tests such as routine blood counts. Fungal testing was performed by scraping the scalp of the alopecia area to obtain scales that were placed on a fluorescence microscope and were negative. Dermoscopy showed broken hair and black dots in the alopecia area, and there were multiple white nodules with different distances in the middle of some hair shafts, with a “brush like” change at the end (Figure 1E,F). Scanning electron microscopy showed that part of the hair cuticle disappeared, and the residual hair cuticle showed a rough and rugged hair shaft (Figure 1G).

Based on the typical nodular changes diagnosed as acquired TN, this patient was advised to reduce the time of scalp massage to 3–5 min after using minoxidil and adjuvant MSC exosomes injection therapy (0.1 mL/cm^2^, 2.5 mL per injection, once a month. Injection method: The injection area was centered on the vertex of the scalp, extending towards the lateral sides. A volume of 0.1 mL was uniformly injected at intervals of 0.5 cm). Avoid physical and chemical damage to the hair during treatment. After 1 month of treatment, the area of hair loss was significantly reduced, hair breakage under dermoscopy was reduced, black dots disappeared, and more new hair could be seen (Figure 1C). After 2 months, the hair loss disappeared, hair breakage under dermoscopy disappeared, and a large amount of new hair could be seen (Figure 1D).

Acquired TN is mostly associated with physicochemical factors and systemic diseases, and common physicochemical injuries include friction, hair coloring and perming, and hair transplantation [1]. It is also associated with iron deficiency and hyper or hypothyroidism [2, 3]. Excessive rubbing of the scalp after minoxidil use in this patient resulted in repeated damage to the hair shaft and thus TN.

There is no standard treatment for this disease, which focuses on avoiding physicochemical injuries to the hair shaft, removing causative factors, and targeting the primary disease [2]. In this study, the use of adipose‐derived mesenchymal stem cell‐derived exosomes (ADSC‐Exos) as an adjuvant treatment for TN has achieved good results. The author's previous study had treated three cases of TN with MSC‐derived exosomes alone, and the hair improved significantly after treatment [4]. Liang et al. showed that ADSC‐Exos promoted hair follicle growth and DPC proliferation and alleviated Dihydrotestosterone inhibition by inhibiting the TGF‐β signaling pathway. miR‐122‐5p regulates the expression of related proteins by targeting SMAD3 to promote the normal growth of hair follicles [5].

## Author Contributions

**Xi Chen:** collection and/or assembly of data, data analysis and interpretation, manuscript writing. **Xinzhou Liu:** data collection, analysis and interpretation. **Jianxin Liu:** treatment procedures and data collection. **Zhenbo Hu:** provision of study material, conception and design. **Guoyan Liu:** the study concept and design, final approval of the final version of the manuscript.

## Ethics Statement

The patients in this manuscript have given written informed consent to the publication of their case details. Hospital for Skin Diseases, Shandong First Medical University, and the treatments were ethically approved by the hospital (Ethics No. 20230709KYKTKS002).

## Conflicts of Interest

The authors declare no conflicts of interest.

## Data Availability

Data sharing is not applicable to this article as no new data were created or analyzed in this study.
